# Association of Thyroid Hormone Concentrations with Levels of Organochlorine Compounds in Cord Blood of Neonates

**DOI:** 10.1289/ehp.10486

**Published:** 2007-09-27

**Authors:** Johan Maervoet, Griet Vermeir, Adrian Covaci, Nicolas Van Larebeke, Gudrun Koppen, Greet Schoeters, Vera Nelen, Willy Baeyens, Paul Schepens, Maria K. Viaene

**Affiliations:** 1 Toxicological Centre, University of Antwerp (UA), Wilrijk, Belgium; 2 Neurotoxicity Expertise Centre, Governmental Psychiatric Hospital, Geel, Belgium; 3 Study Centre for Carcinogenesis and Primary Prevention of Cancer, Ghent University Hospital, Ghent, Belgium; 4 Centre of Expertise in Environmental Toxicology, Flemish Institute of Technological Research (VITO), Mol, Belgium; 5 Provincial Institute for Hygiene, Antwerp, Belgium; 6 Laboratory of Analytical and Environmental Chemistry, Vrije Universiteit Brussel (VUB), Brussels, Belgium; 7 Department of Occupational and Environmental Health, Katholieke Universiteit, Leuven, Leuven, Belgium

**Keywords:** brain, cord blood, development, endocrine disruption, heavy metals, neonates, organohalogens, PCBs, thyroid

## Abstract

**Background:**

Thyroid hormones are important regulators of brain development. During critical periods of development, even transient disorders in thyroid hormone availability may lead to profound neurologic impairment. Animal experiments have shown that certain environmental pollutants, including heavy metals and organochlorine compounds such as polychlorinated biphenyls (PCBs) and dioxins, can interfere with thyroid hormone homeostasis. Whether these contaminants can affect circulating levels of thyroid hormones in humans is unclear, however, because the results of available studies are inconsistent.

**Objectives:**

The aim of the present study is to examine the possible relationships between concentrations of environmental pollutants and thyroid hormone levels in human umbilical cord blood.

**Methods:**

We measured concentrations of environmental pollutants [including selected PCBs, dioxin-like compounds, hexachlorobenzene, *p*,*p*′-DDE (dichlorodiphenyldichloroethylene), cadmium, lead] and thyroid hormones in the cord blood of 198 neonates.

**Results:**

A statistically significant inverse relationship between concentrations of organochlorine compounds and levels of both free triiodothyronine (fT_3_) and free thyroxine (fT_4_), but not thyroid-stimulating hormone, was observed. We found no association between concentrations of heavy metals and thyroid hormone levels.

**Conclusions:**

Our results suggest that environmental chemicals may affect the thyroid system of human neonates. Although the differences in fT_3_ and fT_4_ levels associated with the organochlorine compounds were within the normal range, the observed interferences may still have detrimental effects on the neurologic development of the individual children, given the importance of thyroid hormones in brain development.

It is well established that thyroid hormones are important regulators of brain development during the fetal and neonatal periods of life (Bernal et al. 2003). When maternal and/or fetal thyroid deficiency occurs during pregnancy, the child’s neuropsychological development is adversely affected, and profound and irreversible damage may be caused (Haddow et al. 1999; Morreale de Escobar et al. 2004). Animal experiments have shown that many environmental pollutants, including heavy metals and organohalogen compounds such as polychlorinated biphenyls (PCBs) and dioxins can interfere with the thyroid system through multiple and interactive mechanisms. For instance, they have been reported to alter the structure and size of the thyroid gland, affect thyroid hormone metabolism, and compete with thyroid hormones for transport and carrier molecules (Brouwer et al. 1998; Brucker-Davis 1998; Howdeshell 2002; Zoeller 2005). Moreover, negative associations between developmental exposure to these pollutants and measures of mental, motor, and behavioral functioning have been observed in both animals (reviewed by Mariussen and Fonnum 2006; Schantz and Widholm 2001) and humans (reviewed by Mendola et al. 2002; Roegge and Schantz 2006; Schantz et al. 2003). The observations that *a*) thyroid hormones are essential for normal brain development, *b*) organohalogens can interfere with the thyroid system, and *c*) adverse effects resembling the clinical symptoms of prenatal hypothyroidism were observed after developmental exposure to these compounds have led to the hypothesis that some of the neurotoxic effects of these pollutants could be mediated by the thyroid system (Brouwer et al. 1998; Porterfield 2000; Zoeller 2005).

Laboratory studies have demonstrated that both *ortho*-substituted and dioxin-like PCB congeners can significantly reduce circulating levels of thyroid hormone in animals, which may effectively create a hypothyroid state (Brouwer et al. 1998; Zoeller 2001). In humans, however, the evidence for this is far less convincing. Studies that have investigated the relationship between PCB exposure and thyroid hormone status, in children or adults, have reported a variety of results and observed thyroid hormone levels were generally within normal ranges (Emmett et al. 1988; Koopman-Esseboom et al. 1994; Langer et al. 1998; Longnecker et al. 2000; Meeker et al. 2007; Nagayama et al. 1998; Osius et al. 1999; Sala et al. 2001b; Takser et al. 2005). Nevertheless, many of these studies showed statistically significant differences in circulatory thyroid hormone levels in high-exposure populations compared with low-exposure controls.

In general, scientists who have reviewed the evidence from the literature that relates environmental pollutants to thyroid hormone levels in humans (Boas et al. 2006; Brucker-Davis 1998; Hagmar 2003) consider the results of available studies to be inconclusive. Some animal studies indicate that thyroxine (T_4_) levels in fetal or neonatal serum appear to be more sensitive to PCB exposure than in the adult (Zoeller 2001). Moreover, both human and experimental animal data suggest that the embryo/fetus is more vulnerable to the toxic effects of organohalogen compounds than are adults. For these reasons, we decided to examine the possible relationships between concentrations of environmental pollutants and thyroid hormones in the cord blood of approximately 200 neonates born in selected regions of Flanders. This study is part of a biomonitoring program (2002–2006) that aims to evaluate the impact of the environment on human health in the region of Flanders (Belgium, Europe). This program was commissioned by the Flemish government and coordinated by the Flemish Centre of Expertise for Environment and Health (Brussels, Belgium).

## Materials and Methods

### Subject recruitment and sample collection

The women participating in the study were recruited during their visit at one of 26 maternity hospitals and gave birth between September 2002 and February 2004. All participants signed an informed consent form and had the right to withdraw from the study at any time. The study was approved by the Medical Ethics Committee of the University of Antwerp. In this particular part of the bio-monitoring program, mother–child pairs were recruited from a rural area and three types of industrial areas (near nonferrous smelters, waste incinerators, and the harbors of Antwerp and Ghent). Subjects were included in the study only if they had been living in one of the regions of interest for at least 5 years, delivered at term (36–42 weeks) after an uncomplicated pregnancy, and no major congenital abnormalities or diseases were observed in the newborn. Twins and infants showing abnormal or asymmetrical reflexes during standard neurologic screening were excluded from the study, because the subjects will also be followed in an additional study focusing on neurobehavioral end points. After birth, the umbilical cord was clamped, and approximately 30 mL of cord blood was collected in pre-labeled, EDTA-anticoagulated test tubes from the placental end of the cord. At the hospital’s laboratory, 4 mL of whole blood were transferred to a polypropylene tube for analysis of heavy metals. The remainder of the blood was centrifuged and the plasma was transferred to glass bottles. Three aliquots from each plasma sample were taken for the analysis of dioxin-like activity (5 mL), PCBs and chlorinated pesticides (3 mL), and thyroid hormones and lipid content (1.5 mL).

### Quantification of PCBs and organochlorine pesticides

Because of their reported abundance in cord serum samples, PCB congeners [International Union of Pure and Applied Chemistry (IUPAC) numbers] 118, 138, 153, 170, and 180 were targeted. The organochlorine pesticides (OCPs) under investigation were dichlorodiphenyldichloroethylene (*p,p*′-DDE) and hexachlorobenzene (HCB). We used the procedure for extraction and cleanup of the organochlorine pollutants as described by Covaci and Schepens (2001) with minor modifications. Solvents and other chemicals were obtained from Merck (Darmstadt, Germany). Briefly, an aliquot of the plasma sample was quantitatively transferred to a tube containing an internal standard (PCB-143 in acetone; Dr. Ehrenstorfer GmbH, Augsburg, Germany) and diluted with water. After sonication, formic acid was added as a denaturant. Subsequently, the sample was loaded on a preconditioned C_18_ Empore SPE cartridge. The analytes of interest were eluted onto a cleanup cartridge containing sodium sulfate and acidified silica (44% H_2_SO_4_ w/w) with the purpose of eliminating residual lipids. For the separation and detection of the organochlorine compounds, an HP 6890 gas chromatograph (Hewlett-Packard, Palo Alto, CA, USA) equipped with an HT-8 capillary column was coupled to an HP 5973 mass spectrometer that operated in electron-capture negative ionization mode (Hewlett-Packard). Identification of the analytes was based on the relative retention times and ion chromatograms. For the quantification of the organochlorine compounds, peak area ratios (analyte response/internal standard response) were plotted against the concentration ratios (analyte concentration/internal standard concentration) which were obtained during the creation of linear calibration curves. We assessed external quality control through participation in the Arctic Monitoring and Assessment Program ring test, organized by the Toxicological Center of Québec, Canada. The limit of quantification (LQ) was 0.02 ng/mL for all organochlorine analytes. Measurements below this limit were corrected to a value equal to half of the quantification limit.

### CALUX bioassay

Whereas the analysis of the individual PCB congeners provides information about cord blood concentrations of non-dioxin-like congeners, the Chemical-Activated LUciferase gene eXpression bio-assay (CALUX; BioDetection Systems BV, Amsterdam, the Netherlands) is a useful screening tool for assessing the newborn’s exposure to dioxin-like compounds. Dioxins and related compounds, including dioxin-like PCBs and furans, elicit a number of common biochemical and toxic responses that are mediated primarily by interaction with a specific cellular protein known as the aryl hydrocarbon (Ah) receptor (Denison and Heath-Pagliuso 1998). In the CALUX assay (BioDetection Systems BV), the dioxin-like activity of the compounds in a sample is assessed via *in vitro* activation of the Ah receptor of cultured H4IIE cells. When exposed to dioxin-like compounds, the recombinant cells express fire-fly luciferase genes and luminesce after the addition of luciferin. The method involved *n*-hexane extraction of the cord blood sample and removal of matrix components by passage through an acidified silica column (33% H_2_SO_4_ w/w). Subsequently, we calculated CALUX-based toxicity equivalents (TEQs) by comparing the luciferase activity induced by the sample extract to a dose–response curve generated from 2,3,7,8-tetrachlorodibenzo-*p*-dioxin concentration standards. The CALUX bioassay (BioDetection Systems BV) is described elsewhere in further detail (Koppen et al. 2001; Murk et al. 1998). The limit of detection was calculated as the light signal measured from the dimethylsulfoxide solvent control on each well plate plus three times its standard deviation: 0.13 ± 0.04 pg CALUX-TEQ/g lipid. On each well plate, a control plasma sample was included for internal quality control purposes.

### Lipid determination

Plasma total lipid (TL) concentrations were determined gravimetrically during execution of the CALUX bioassay (BioDetection Systems BV). Additionally, triglyceride (TG) and cholesterol (CH) levels were measured individually by spectrophotometry on a modular analyzer (Roche Diagnostics, Basel, Switzerland) at the medical laboratory Algemeen Medisch Labo in Antwerp. Detection limits were 4 mg/dL and 3 mg/dL for TG and CH, respectively. The combination of both methods allowed for the creation of a gravimetrically derived formula using concentrations of total cholesterol and triglycerides: TL = 1.33 × (TG + CH) + 50.5 mg/dL. When no value could be obtained gravimetrically, this formula was used to estimate plasma total lipid concentrations.

### Analysis of heavy metals

Additionally, we determined lead and cadmium concentrations in the cord blood samples. During sample preparation, 200 μL of whole blood was chemically digested with a 2-mL mixture of nitric acid and water peroxide (1:1) and heated at 120°C to destroy the blood matrix and liberate the metals. After dilution with milli-Q water, 1 ppb of ^115^Indium was added as an internal standard. For the detection and quantification, a high resolution inductively coupled plasma mass spectrometer (Element 2; ThermoFinnigan, Bremen, Germany) was used. Detection limits for cadmium (^114^Cd) and lead (^208^Pb) in blood were 0.09 and 2.0 ng/mL, respectively. Measurements below this limit were corrected to a value equal to half of the detection limit.

### Thyroid hormone analysis

We determined cord blood concentrations of free 3,5,3′-tri-iodothyronine (fT_3_), free thyroxine (fT_4_), and thyroid-stimulating hormone (TSH) by direct chemiluminescence immunoassay on an ADVIA Centaur analyzer (Siemens Medical Solutions Diagnostics, Tarrytown, NY, USA) at the medical laboratory Algemeen Medisch Labo in Antwerp. Whereas the ADVIA Centaur FT3 and FrT4 assays are labeled antibody methods involving competitive immunoassay, the TSH-3 assay is a two-site sandwich method. Laboratory reference values for adults range from 3.5 to 6.5 pmol/L for fT_3_, from 10.2 to 24.6 pmol/L for fT_4_, and from 0.35 to 5.5 mIU/L for TSH. For neonates younger than 1 day, the laboratory’s reference values for TSH range from 11.6 to 35.9 mIU/L.

### Statistical methods

We calculated Spearman’s rank correlation coefficients (*r*_s_) to assess the relationships between fT_3_, fT_4_, and TSH levels in the cord blood, as well as between the concentrations of the different pollutants. We used linear regression analysis to evaluate the association of cord blood concentrations of the contaminants with thyroid hormone levels. Thyroid hormone values were transformed by natural logarithm (ln) to obtain variables that were approximately normally distributed. Each of the contaminants was analyzed as a predictor in separate regression models because a high degree of inter-correlation is typically observed between concentrations of individual PCBs and other organochlorine compounds. It is clear that the lipid content may influence plasma concentrations of liphophilic pollutants such as PCBs and OCPs. Whereas some scientists express measurements as lipid-standardized values by dividing the wet weight concentration (e.g., PCB per unit of plasma) by plasma lipids, others include plasma lipids as a separate term in the regression equation. In the present study, plasma lipids are included as a separate predictor in the regression models because it was recently shown that lipid standardization models are highly prone to bias (Schisterman et al. 2005). The regression models were also adjusted for four potentially confounding factors: gestational age, sex, alcohol consumption during pregnancy, and age of the mother. Reported regression coefficients are unstandardized coefficients, and *p*-values < 0.05 indicate statistically significant differences.

## Results

### Study population

The women participating in this study (*n* = 198) averaged 29.4 years of age (range, 20–42 years) at the time they gave birth. About 14% of them smoked during pregnancy, whereas 6% consumed alcohol. On average, births occurred after 39.2 weeks of gestation. Ninety-seven infants (49%) were male and 101 (51%) were female. The newborns had a mean birth weight of 3.49 kg (range, 2.34–5.58 kg) and body height of 50.5 cm (range, 45.0–57.0 cm).

### Hormone levels

Means, standard deviations, medians, and 5^th^–95^th^ percentiles of the cord blood thyroid hormone levels are presented in Table 1. A strong positive correlation was observed between fT_3_ and fT_4_ levels (*r*_s_ = 0.52; *p* < 0.0001), whereas a weak positive correlation was found between levels of fT_4_ and TSH (*r*_s_ = 0.19; *p* < 0.01). Six infants had TSH cord blood levels > 25 mIU/L, but none were diagnosed with hypothyroidism or any other thyroid-related condition.

### Contaminant levels

Concentrations of PCBs, OCPs, and dioxin-like compounds detected in the cord blood samples are summarized in Table 2. Both wet weight and lipid-adjusted concentrations are shown in order to facilitate comparison with values reported in the literature. Concentrations of plasma TL averaged 213 ± 75 mg/dL (mean ± SD; range, 52–614 mg/dL). As is typical for this type of epidemiologic study, medium to high positive correlation coefficients (*r*_s_ = 0.22 to 0.86) were observed between the cord blood concentrations of nearly all individual organochlorine compounds (results not shown). Only CALUX-TEQ values were not significantly correlated with levels of *p,p*′-DDE, PCB-153, PCB-170, or PCB-180.

With regard to the heavy metals, mean cord blood concentrations of cadmium and lead (both *n* = 189) were determined at 0.5 ± 1.1 ng/mL [5^th^–95^th^ percentile, BLQ (below limit of quantification)–1.9 ng/mL] and 19.4 ± 15.8 ng/mL (5^th^–95^th^ percentile, 2.9–53.1 ng/mL), respectively. Lead concentrations never exceeded the blood level of concern of 100 ng/mL set by the Centers for Disease Control and Prevention in 1991 (Szpir 2006). We identified a statistically significant positive correlation between cord blood concentrations of cadmium and lead (*r*_s_ = 0.47; *p* < 0.0001). No significant correlations were observed between concentrations of the heavy metals and any individual organochlorine compound.

### Association of thyroid hormone concentrations with levels of environmental contaminants in cord blood

**Figure 1:**
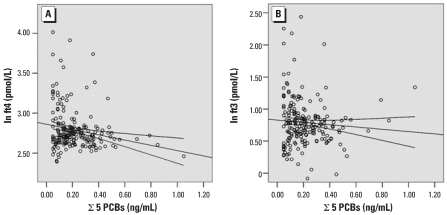
Scatterplots showing the negative association of PCB concentrations (sum of 5 congeners) with fT_4_ (*A*) and fT_3_ (*B*) levels in human cord blood. The curves represent the 95% confidence interval of the fit line.

Table 3 shows the results of the multiple linear regression analyses that were run to investigate the possible association of cord blood thyroid hormone concentrations with levels of the different contaminants. We observed a strong inverse relationship between levels of all of the individual organohalogen compounds and concentrations of fT_4_ (Table 3). A somewhat weaker, yet still statistically significant, inverse association of fT_3_ levels with concentrations of PCBs 118, 138, 170, 180, HCB, and the CALUX-TEQ values was identified. Scatterplots showing the inverse association of cord blood PCB concentrations (sum of five congeners) with (ln-transformed) levels of fT_4_ (β = −0.59; SE = 0.13; *p* < 0.001) and fT_3_ (β = −0.48; SE = 0.19; *p* = 0.01) are presented in Figure 1. In contrast, TSH levels were not related to the concentrations of any of the contaminants (Table 3). Furthermore, no relationship between exposure to heavy metals and thyroid hormone levels was identified.

## Discussion

Our results reveal statistically significant inverse associations of umbilical cord blood concentrations of organochlorine compounds with fT_3_ and fT_4_ but not TSH levels. Similar observations were made in experimental animals after exposure to individual PCB congeners, commercial PCB mixtures, and dioxins. In general, laboratory studies demonstrate that PCBs and related compounds can profoundly decrease circulating levels of T_4_ while having little or no effect on T_3_ and TSH concentrations (Brouwer et al. 1998; Zoeller 2001). Whether exposure to these compounds can affect human thyroid hormone homeostasis is unclear, however, because available studies yielded conflicting results (reviewed by Boas et al. 2006; Hagmar 2003). Whereas some scientists reported relationships between levels of organohalogens and thyroid hormones in humans (Hagmar et al. 2001b; Koopman-Esseboom et al. 1994; Meeker et al. 2007; Nagayama et al. 1998; Osius et al. 1999; Persky et al. 2001; Sala et al. 2001b; Schell et al. 2004; Takser et al. 2005), others found no such associations (Fiolet et al. 1997; Hagmar et al. 2001a; Longnecker et al. 2000; Matsuura et al. 2001; Steuerwald et al. 2000).

Fetuses and neonates are believed to be more vulnerable to the effects of PCBs and related compounds than adults. In 1968 and 1978–1979, two mass poisoning incidents, each involving about 2,000 people, occurred in Japan (Yusho) and Taiwan (Yu-Cheng), respectively. In both cases, people had ingested rice bran oil that had been accidentally contaminated with thermally degraded PCBs during the manufacturing process (Aoki 2001). Children born to mothers who consumed this oil exhibited various physical and behavioral deficits including hypothyroidism and developmental delay (Zoeller 2001). More recently, several epidemiologic studies have reported negative associations between prenatal exposure to background levels of PCBs and measures of cognitive and/or motor functioning in infancy or childhood (reviewed by Roegge and Schantz 2006; Schantz et al. 2003). There is some evidence supporting the hypothesis that organochlorine pollutants may exert such adverse effects on the developing brain by causing a state of relative hypothyroidism (Zoeller 2005). From the literature, we identified 14 studies in which the effects of background exposure to PCBs and related compounds on the thyroid hormone status of human neonates/infants and pregnant women were investigated (Table 4). Results of these studies are conflicting and certainly do not convincingly show that organohalogen compounds can affect thyroid hormone concentrations in these target groups. To our knowledge, the present study is the first demonstrating such clear and consistent associations between exposure to PCBs/dioxins and levels of thyroid hormones in human neonates.

The fact that it is very difficult to standardize the design of this kind of epidemiologic study may, at least in part, explain the conflicting results reported in the literature. First, researchers have used a number of different matrices for quantifying the child’s level of exposure to organohalogen compounds which include breast milk, placental tissue, umbilical cord blood, and serum of both the mother and the infant. Whereas a lipid-rich substance such as breast milk was commonly used in the 1990s, modern analytical techniques allow detection and accurate quantification of PCBs and related compounds in a low-fat matrix such as umbilical cord blood. Second, many individual compounds are suspected of interfering with the thyroid system (Brucker-Davis 1998), and often a strong degree of collinearity is observed among environmental levels of many of these pollutants. Obviously, this makes it very difficult to determine which of the compounds may actually have an effect on human thyroid hormone levels. Although dioxin-like compounds, pesticides, and PCBs almost certainly act via multiple mechanisms of action (Brouwer et al. 1998; Brucker-Davis 1998; Howdeshell 2002), it is believed that their thyroid hormone–disrupting effects may in fact be cumulative or synergistic (Crofton et al. 2005). Furthermore, it is known that organohalogen compounds were most frequently used in the northern hemisphere and that environmental levels are gradually declining (Dallaire et al. 2003). Therefore, variation in exposure levels in different geographic regions and over time may also complicate comparison of study results. In the present study, PCB concentrations are at the lower end of the range reported in earlier studies (Table 5). So it seems unlikely that the observed results can be attributed to local, elevated levels of exposure. Third, although the thyroid hormone system is controlled by a feedback mechanism and concentrations of the thyroid hormones are interrelated, so far no researchers have quantified all possible iodothyronines. For instance, only Steuerwald et al. (2000) previously measured concentrations of fT_3_, the free form of the biologically most active thyroid hormone triiodothyronine. Finally, various and often unclear strategies for confounder adjustments were used in the different studies. As a result, we believe it is difficult to compare the results of the available studies (Table 4) because so many parameters, including the exposure and outcome variables, are varying.

The cord blood concentrations of thyroid hormones reported in the present study are similar to values published by other authors (Henry et al. 2000; Hume et al. 2004; Morreale de Escobar et al. 2004; Williams et al. 2004). It is clear that the detected fT_3_ and TSH levels (Table 1) are different from the laboratory’s reference values (see “Materials and Methods”). This observation can be explained by the fact that the thyroid hormones present in cord blood are both of maternal and fetal origin. Whereas fetal fT_4_ serum levels are similar to maternal values at term, fT_3_ concentrations are 2- to 3-fold lower and fetal circulating TSH levels are much higher than maternal and adult values (Howdeshell 2002; Morreale de Escobar et al. 2004). We observed an inverse relationship between cord blood levels of the organochlorine compounds and concentrations of both fT_3_ and fT_4_ but not TSH (Table 3). This is in accordance with earlier studies that showed that PCBs can cause severe hypothyroxinemia and attenuate the anticipated TSH response in experimental animals (Brouwer et al. 1998; Khan and Hansen 2003). It is important to recognize that the differences in fT_3_ and fT_4_ levels associated with the organohalogen compounds in the present study were still within the normal range. Our results suggest that a 0.5-ng/mL increase in cord blood PCB levels (sum of five congeners) may be associated with a decrease of about 0.5 pmol/L of fT_3_ and 3.5 pmol/L of fT_4_. Statistically speaking, the organohalogens explained up to 13% of the variance in the linear regression models. Because there is no evidence for background exposure to these compounds causing overt hypothyroidism in humans as it does in experimental animals, some scientists may argue that, despite being statistically significant, such findings are clinically irrelevant (Kimbrough and Krouskas 2001). However, others believe that even very subtle changes in the T_4_ and TSH homeostasis may affect the development of the human fetus (Boas et al. 2006; Zoeller 2001). In their recent review article, Boas et al. (2006) explained that the T_4_/TSH relationship is very unique for each person, and that the intraindividual variation of thyroid hormone levels is small compared with the population-based reference intervals. Therefore, small changes in thyroid function within the normal reference range may still have negative health consequences for individuals. Because the fetal turnover of T_4_ is very rapid, the human fetus may be particularly vulnerable.

Because it is relatively easy to measure thyroid hormone concentrations, most studies to date use circulating levels of thyroid hormone as the primary indicator of an effect on the thyroid system, or focus on mechanisms by which contaminants could affect thyroid hormone levels. Yet an increasing number of reports are revealing that environmental pollutants may also interfere with the thyroid system at several other interaction sites. For instance, they may bind to thyroid hormone receptors, influence the interaction of these receptors with various co-factors or cause them to exhibit a different affinity for the thyroid hormone–responsive elements on DNA (Zoeller 2005). Therefore, one must consider that organohalogen compounds may not only affect circulating levels of thyroid hormones, but may also interfere with thyroid hormone–regulated processes further downstream, such as signaling and gene expression.

In conclusion, analysis of the umbilical cord blood of nearly 200 neonates revealed a statistically significant, inverse relationship between concentrations of organochlorines and levels of both fT_3_ and fT_4_ but not TSH. These findings provide evidence for the hypothesis that background exposure to environmental chemicals may affect the thyroid system in human neonates. There are still many gaps in our understanding of the mechanisms by which environmental pollutants may interact with the thyroid system. It is clear, however, that thyroid hormones are important regulators of brain development, and research suggests that such interferences may adversely affect neurodevelopment early in life, which in turn may have long-term neurologic consequences for individuals.

## Figures and Tables

**Table 1:** Thyroid hormone concentrations measured in umbilical cord blood.

Hormone	No.	Mean	SD	Median	5th percentile	95th percentile
fT_3_ (pmol/L)	197	2.4	1.3	2.1	1.3	4.0
fT_4_ (pmol/L)	198	17.0	6.1	15.2	12.5	27.7
TSH (mIU/L)	198	8.2	5.5	6.6	2.8	17.8

**Table 2:** Concentrations of PCBs, organochlorine pesticides, and dioxin-like compounds measured in cord blood.

		Wet weight concentrations (ng/mL plasma)^a^					Lipid adjusted concentrations (ng/g lipid)^a^				
Contaminant	No.	Mean	SD	Median	5th percentile	95th percentile	Mean	SD	Median	5th percentile	95th percentile
PCBs											
PCB-118	198	0.03	0.02	0.02	BLQ	0.07	14.3	11.2	10.9	3.3	36.6
PCB-138	198	0.04	0.04	0.03	BLQ	0.10	20.8	16.3	16.9	3.5	56.3
PCB-153	198	0.07	0.06	0.06	BLQ	0.19	37.1	28.9	31.7	3.8	104
PCB-170	198	0.02	0.01	BLQ	BLQ	0.04	9.1	6.9	6.5	3.2	23.7
PCB-180	198	0.05	0.03	0.04	BLQ	0.11	25.0	16.4	23.3	4.0	57.5
∑5 PCBs	198	0.21	0.15	0.18	BLQ	0.49	106	72.2	91.7	20.0	256
OCPs											
HCB	198	0.05	0.04	0.04	BLQ	0.13	25.9	20.7	22.4	3.7	67.3
*p,p*′-DDE	198	0.37	0.36	0.27	0.05	1.11	189	193	134	25.3	628
Dioxin-like compounds											
CALUX-TEQ^a^	140	0.06	0.03	0.05	0.02	0.12	30.6	20.6	26.3	7.4	64.9

BLQ, below limit of quantification.

a CALUX-TEQ wet-weight values are expressed in picograms per milliliter, whereas lipid-adjusted values are expressed in picograms per gram.

**Table 3:** Unstandardized regression coefficients reflecting the relationship between thyroid hormone concentrations and levels of environmental contaminants in cord blood.

	ln fT_3_ (pmol/L)				ln fT_4_ (pmol/L)				ln TSH (mIU/L)			
	B	SE	*p-*Value	No.	B	SE	*p-*Value	No.	B	SE	*p*-Value	No.
PCBs												
PCB-118 (ng/mL)	−3.45	1.19	< 0.01	195	−3.96	0.83	< 0.001	196	−1.71	1.93	0.38	196
PCB-138 (ng/mL)	−2.56	0.75	< 0.001	195	−2.97	0.51	< 0.001	196	−1.72	1.23	0.16	196
PCB-153 (ng/mL)	−0.63	0.45	0.17	195	−0.93	0.32	< 0.01	196	0.01	0.73	0.99	196
PCB-170 (ng/mL)	−5.22	2.15	0.02	195	−6.90	1.48	< 0.001	196	−4.14	3.45	0.23	196
PCB-180 (ng/mL)	−2.19	0.88	0.01	195	−2.35	0.62	< 0.001	196	−0.62	1.42	0.66	196
∑5 PCBs (ng/mL)	−0.48	0.19	0.01	195	−0.59	0.13	< 0.001	196	−0.20	0.30	0.50	196
OCPs												
HCB (ng/mL)	−1.47	0.68	0.03	195	−1.92	0.47	< 0.001	196	−0.87	1.08	0.42	196
*p,p*′-DDE (ng/mL)	−0.07	0.07	0.29	195	−0.10	0.05	0.04	196	−0.07	0.11	0.51	196
Dioxin-like compounds												
CALUX-TEQ (pg/mL)	−1.66	0.81	0.04	138	−1.30	0.62	0.04	138	−0.29	1.36	0.83	138
Heavy metals												
Cadmium (ng/mL)	−0.04	0.04	0.23	186	−0.02	0.03	0.58	187	−0.03	0.07	0.62	187
Lead (ng/mL)	−0.00	0.00	0.15	186	0.00	0.00	0.37	187	0.00	0.00	0.57	187

B, unstandardized regression coefficient. Each of the contaminants was used as a predictor in a separate linear regression model. All models were adjusted for plasma total lipids, gestational age, sex, alcohol consumption during pregnancy, and age of the mother.

**Table 4:** Association between exposure to organohalogen compounds and concentrations of thyroid hormones in human neonates/infants and pregnant women (adapted from Hagmar 2003).

		Pollutants		Thyroid hormones						
Study	No.	Exposure matrix - Biomarkers	Sampling time	TT_3_	fT_3_	TT_4_	fT_4_	TSH	Matrix	Sampling time
Pregnant women												
Koopman-Esseboom et al. 1994	78	Breast milk TEQ	2nd week after delivery	↓		↓	→	→	Maternal plasma	2nd week after delivery
Steuerwald et al. 2000	173	Maternal serum PCBs & OCPs	Week 34 of gestation		→	→	→	→	Maternal serum	At birth
Takser et al. 2005	149	Maternal plasma ∑PCBs	During pregnancy	↓			→	→	Maternal plasma	During pregnancy
Infants and neonates												
Pluim et al. 1993	38	Breast milk TEQ	3rd week after delivery			↑		→	Infant plasma	1 week after delivery
		Breast milk TEQ	3rd week after delivery			↑		↑	Infant plasma	11 weeks after delivery
Koopman-Esseboom et al. 1994	78	Breast milk TEQ	2nd week after delivery	→		→	→	→	Cord plasma	At birth
		Breast milk TEQ	2nd week after delivery	→		→	→	↑	Infant plasma	2nd week and 3rd month
Fiolet et al. 1997	93	Breast milk PCBs, PCDD/Fs, OCPs	2nd week after delivery			→		→	Infant plasma	1st week after delivery
Nagayama et al. 1997	71	Breast milk TEQ	2–3 months after delivery	→		↓		↑	Infant plasma	1 year after delivery
Nagayama et al. 1998	36	Breast milk TEQ	3 months after delivery	↓		↓		→	Infant plasma	1 year after delivery
Longnecker et al. 2000	160	Breast milk and maternal serum, Total PCBs (Webb-McCall method)	Postpartum period			→	→	→	Cord serum	At birth (1978–1982)
Steuerwald et al. 2000	173	Maternal serum PCBs & OCPs	Week 34 of gestation		→	→	→	→	Cord serum	At birth
Matsuura et al. 2001	337	Breast milk TEQ	30 days after delivery					→	Infant serum	1 year after delivery
Sandau et al. 2002	20	Cord plasma ∑PCBs	At birth	→			→	↓	Cord plasma	At birth
	20	Cord plasma PCP + ∑HO-PCBs	At birth	↓			↓	→	Cord plasma	At birth
Ribas-Fitó et al. 2003	70	Cord serum PCBs and HCB	At birth					→	Infant plasma	3 days after delivery
Takser et al. 2005	92	Cord blood ∑PCBs	At birth	→			→	→	Cord blood	At birth
Wang et al. 2005	118	Placenta TEQ		→		→	→	→	Cord blood	At birth
Asawasinsopon et al. 2006	39	Cord serum OCPs	At birth			↓	→	→	Cord serum	At birth
Chevrier et al. 2007	285	Maternal serum ∑PCBs, TEQ	2nd trimester					→	Infant plasma	At birth
		CYP2B-inducing PCBs	2nd trimester					↑	Infant plasma	At birth
Present study	198	Cord plasma ∑5 PCBs	At birth		↓		↓	→	Cord plasma	At birth
		Cord plasma TEQ	At birth		↓		↓	→	Cord plasma	At birth

→ no association; ↑ positive association; ↓ negative association.

**Table 5:** Comparison of mean concentrations (ng/mL) of PCBs and organochlorine pesticides measured in umbilical cord blood samples reported in the present and earlier studies.

Region	Samples	HCB	*p,p*′-DDE	∑PCBs	Reference
Flanders	198	0.05	0.37	0.20^a^	Present study
Faroe Islands	316	—	1.76	1.36^a^	Barr et al. 2006
Spain	405	0.68	1.03	0.69^b^	Sunyer et al. 2005
Canada	92	—	—	0.05^c^	Takser et al. 2005
The Netherlands	51	—	—	0.48^a^	Soechitram et al. 2004
Arctic Canada	400	0.10	0.53	0.26^a^	Walker et al. 2003
Belgium	44	—	0.58	0.47^a^	Covaci et al. 2002
Spain	69	1.11	0.83	0.23^a^	Sala et al. 2001a
Germany	171	—	—	0.39^c^	Walkowiak et al. 2001
United States	751	0.03	0.48	0.25^a^	Korrick et al. 2000
Canada	1,109	—	0.41	0.51^d^	Rhainds et al. 1999
The Netherlands	415	—	—	0.38^a^	Huisman et al. 1995

a Sum of PCBs 118, 138, 153, and 180.

b Sum of PCBs 28, 52, 101, 118, 153, 138, and 180.

c Sum of PCBs 138, 153, and 180.

d Sum PCBs expressed as Aroclor 1260.

## References

[b1-ehp0115-001780] Aoki Y (2001). Polychlorinated biphenyls, polychlorinated dibenzo-*p*-dioxins, and polychlorinated dibenzofurans as endocrine disrupters—what we have learned from Yusho disease. Environ Res Section A.

[b2-ehp0115-001780] Asawasinsopon R, Prapamontol T, Prakobvitayakit O, Vaneesorn Y, Mangklabruks A, Hock B (2006). The association between organochlorine and thyroid hormone levels in cord serum: a study from northern Thailand. Environ Int.

[b3-ehp0115-001780] Barr DB, Weihe P, Davis MD, Needham LL, Grandjean P (2006). Serum polychlorinated biphenyl and organochlorine insecticide concentrations in a Faroese birth cohort. Chemosphere.

[b4-ehp0115-001780] Bernal J, Guaduaño-Ferraz A, Morte B (2003). Perspectives in the study of thyroid hormone action on brain development and function. Thyroid.

[b5-ehp0115-001780] Boas M, Feldt-Rasmussen U, Skakkebæk NE, Main KM (2006). Environmental chemicals and thyroid function. Eur J Endocrinol.

[b6-ehp0115-001780] Brouwer A, Morse DC, Lans MC, Schuur AG, Murk AJ, Klasson-Wehler E (1998). Interactions of persistent environmental organohalogens with the thyroid hormone system: mechanisms and possible consequences for animal and human health. Toxicol Ind Health.

[b7-ehp0115-001780] Brucker-Davis F (1998). Effects of environmental synthetic chemicals on thyroid function. Thyroid.

[b8-ehp0115-001780] Chevrier J, Eskenazi B, Bradman A, Fenster L, Barr DB (2007). Associations between prenatal exposure to polychlorinated biphenyls and neonatal thyroid-stimulating hormone levels in a Mexican-American population, Salinas Valley, California. Environ Health Perspect.

[b9-ehp0115-001780] Covaci A, Jorens P, Jacquemyn Y, Schepens P (2002). Distribution of PCBs and organochlorine pesticides in umbilical cord and maternal serum. Sci Total Environ.

[b10-ehp0115-001780] Covaci A, Schepens P (2001). Improved determination of selected POPs in human serum by solid phase disk extraction and GC-MS. Chemosphere.

[b11-ehp0115-001780] Crofton KM, Craft ES, Hedge JM, Gennings C, Simmons JE, Carchman RA (2005). Thyroid-hormone–disrupting chemicals: evidence for dose-dependent additivity or synergism. Environ Health Perspect.

[b12-ehp0115-001780] Dallaire F, Dewailly É, Muckle G, Ayotte P (2003). Time trends of persistent organic pollutants and heavy metals in umbilical cord blood of Inuit infants born in Nunavik (Québec, Canada) between 1994 and 2001. Environ Health Perspect.

[b13-ehp0115-001780] Denison MS, Heath-Pagliuso S (1998). The Ah receptor: a regulator of the biochemical and toxicological actions of structurally diverse chemicals. Bull Environ Contam Toxicol.

[b14-ehp0115-001780] Emmett EA, Maroni M, Jeffreys J, Schmith J, Levin BK, Alvares A (1988). Studies of transformer repair workers exposed to PCBs: II. Results of clinical laboratory investigations. Am J Indust Med.

[b15-ehp0115-001780] Fiolet DCM, Cuijpers CEJ, Lebret E (1997). Exposure to polychlorinated organic compounds and thyroid hormone plasma levels of human newborns. Organohalogen Compounds.

[b16-ehp0115-001780] Haddow JE, Palomaki GE, Allen WC, Williams JR, Knight GJ, Gagnon J (1999). Maternal thyroid deficiency during pregnancy and subsequent neuropsychological development of the child. N Engl J Med.

[b17-ehp0115-001780] Hagmar L (2003). Polychlorinated biphenyls and thyroid status in humans: a review. Thyroid.

[b18-ehp0115-001780] Hagmar L, Björk J, Sjödin A, Bergman Å, Erfurth EM (2001a). Plasma levels of persistent organohalogens and hormone levels in adult male humans. Arch Environ Health.

[b19-ehp0115-001780] Hagmar L, Rylander L, Dyremark E, Klasson-Wehler E, Bergman Å, Erfurth EM (2001b). Plasma concentrations of persistent organochlorines in relation to thyrotropin and thyroid hormone levels in women. Int Arch Occup Environ Health.

[b20-ehp0115-001780] Henry JG, Sobki SH, Al Beshara NM, Harkonen ME, Miller HR (2000). Thyroid function in cord blood. Saudi Med J.

[b21-ehp0115-001780] Howdeshell KL (2002). A model of the development of the brain as a construct of the thyroid system. Environ Health Perspect.

[b22-ehp0115-001780] Huisman M, Koopman-Esseboom C, Fidler V, Hadders-Algra M, Van der Paauw CG, Tuinstra LGM (1995). Perinatal exposure to polychlorinated biphenyls and dioxins and its effect on neonatal neurological development. Early Hum Dev.

[b23-ehp0115-001780] Hume R, Simpson J, Delahunty C, van Toor H, Wu SY, Williams FLR (2004). Human fetal and cord serum thyroid hormones: developmental trends and interrelationships. J Clin Endocrinol Metab.

[b24-ehp0115-001780] Khan MA, Hansen LG (2003). *ortho*-Substituted polychlorinated biphenyl (PCB) congeners (95 or 101) decrease pituitary response to thyrotropin releasing hormone. Toxicol Lett.

[b25-ehp0115-001780] Kimbrough RD, Krouskas CA (2001). Polychlorinated biphenyls, dibenzo-*p*-dioxins, and dibenzofurans and birth weight and immune and thyroid function in children. Regul Toxicol Pharmacol.

[b26-ehp0115-001780] Koopman-Esseboom C, Morse DC, Weisglas-Kuperus N, Lutkeschipholt IJ, Van der Paauw CG, Tuinstra LGMT (1994). Effects of dioxins and polychlorinated biphenyls on thyroid hormone status of pregnant women and their infants. Pediatr Res.

[b27-ehp0115-001780] Koppen G, Covaci A, Van Cleuvenbergen R, Schepens P, Winneke G, Nelen V (2001). Comparison of CALUX-TEQ values with PCB and PCDD/F measurements in human serum of the Flanders Environmental and Health Study (FLEHS). Toxicol Lett.

[b28-ehp0115-001780] Korrick SA, Altshul LM, Tolbert PE, Burse VW, Needham LL, Monson RR (2000). Measurement of PCBs, DDE, and hexachlorobenzene in cord blood from infants born in towns adjacent to a PCB contaminated waste site. J Expo Anal Environ Epidemiol.

[b29-ehp0115-001780] Langer P, Tajtakova M, Fodor G, Kocan A, Bohov P, Michalek J (1998). Increased thyroid volume and prevalence of thyroid disorders in an area heavily polluted by polychlorinated biphenyls. Eur J Endocrinol.

[b30-ehp0115-001780] Longnecker MP, Gladen BC, Patterson DG, Rogan WJ (2000). Polychlorinated biphenyl (PCB) exposure in relation to thyroid hormone levels in neonates. Epidemiology.

[b31-ehp0115-001780] Mariussen E, Fonnum F (2006). Neurochemical targets and behavioral effects of organohalogen compounds: an update. Crit Rev Toxicol.

[b32-ehp0115-001780] Matsuura N, Uchiyama T, Tada H, Nakamura Y, Kondo N, Morita M (2001). Effects of dioxins and polychlorinated biphenyls (PCBs) on thyroid function in infants born in Japan—the second report from research on environmental health. Chemosphere.

[b33-ehp0115-001780] Meeker JD, Altshul L, Hauser R (2007). Serum PCBs, *p,p*′-DDE and HCB predict thyroid hormone levels in men. Environ Res.

[b34-ehp0115-001780] Mendola P, Selevan SG, Gutter S, Rice D (2002). Environmental factors associated with a spectrum of neurodevelopmental deficits. Ment Retard Dev Disabil Res Rev.

[b35-ehp0115-001780] Morreale de Escobar G, Obregón MJ, Escobar del Rey F (2004). Maternal thyroid hormones early in pregnancy and fetal brain development. Best Pract Res Clin Endocrinol Metab.

[b36-ehp0115-001780] Murk AJ, Leonards PEG, van Hattum B, Luit R, van der Weiden MEJ, Smit M (1998). Application of biomarkers for exposure and effect of polyhalogenated aromatic hydrocarbons in naturally exposed European otters (*Lutra lutra*). Environ Toxicol Pharmacol.

[b37-ehp0115-001780] Nagayama J, Iida T, Hirakawa H, Matsueda T, Okamura K, Hasegawa M (1997). Effects of lactational exposure to chlorinated dioxins and related chemicals on thyroid function in Japanese babies. Organohalogen Compounds.

[b38-ehp0115-001780] Nagayama J, Okamura K, Iida T, Hirakawa H, Matsueda T, Tsuji H (1998). Postnatal exposure to chlorinated dioxins and related chemicals on thyroid hormone status in Japanese breast-fed infants. Chemosphere.

[b39-ehp0115-001780] Osius N, Karmaus W, Kruse H, Witten J (1999). Exposure to polychlorinated biphenyls and levels of thyroid hormones in children. Environ Health Perspect.

[b40-ehp0115-001780] Persky V, Turyk M, Anderson HA, Hanrahan LP, Falk C, Steenport DN (2001). The effects of PCB exposure and fish consumption on endogenous hormones. Environ Health Perspect.

[b41-ehp0115-001780] Pluim HJ, de Vijlder JJ, Olie K, Kok JH, Vulsma T, van Tijn DA (1993). Effects of pre- and postnatal exposure to chlorinated dioxins and furans on human neonatal thyroid hormone concentrations. Environ Health Perspect.

[b42-ehp0115-001780] Porterfield SP (2000). Thyroidal dysfunction and environmental chemicals: potential impact on brain development. Environ Health Perspect.

[b43-ehp0115-001780] Rhainds M, Levallois P, Dewailly É, Ayotte P (1999). Lead, mercury, and organochlorine compound levels in cord blood in Québec, Canada. Arch Environ Health.

[b44-ehp0115-001780] Ribas-Fitó N, Sala M, Cardo E, Mazón C, De Muga ME, Verdú A (2003). Organochlorine compounds and concentrations of thyroid stimulating hormone in newborns. Occup Environ Med.

[b45-ehp0115-001780] Roegge CJ, Schantz SL (2006). Motor function following developmental exposure to PCBs and/or MeHg. Neurotoxicol Teratol.

[b46-ehp0115-001780] Sala M, Ribas-Fitò N, Cardo E, de Muga ME, Marco E, Mazón C (2001a). Levels of hexachorobenzene and other organochlorine compounds in cord blood: exposure across placenta. Chemosphere.

[b47-ehp0115-001780] Sala M, Sunyer J, Herrero C, To-Figueras J, Grimalt J (2001b). Association between serum concentrations of hexachlorobenzene and polychlorobiphenyls with thyroid hormone and liver enzyme in a sample of the general population. Occup Environ Med.

[b48-ehp0115-001780] Sandau CD, Ayotte P, Dewailly É, Duffe J, Norstrom RJ (2002). Pentachlorophenol and hydroxylated polychlorinated biphenyl metabolites in umbilical cord plasma of neonates from coastal populations in Québec. Environ Health Perspect.

[b49-ehp0115-001780] Schantz SL, Widholm JJ, Robertson LW, Hansen LG (2001). Effects of PCB exposure on neurobehavioral function in animal models. PCBs: Recent Advances in Environmental Toxicology and Health Effects.

[b50-ehp0115-001780] Schantz SL, Widholm JJ, Rice DC (2003). Effects of PCB exposure on neuropsychological function in children. Environ Health Perspect.

[b51-ehp0115-001780] Schell LM, Gallo MV, DeCaprio AP, Hubicki L, Denham M, Ravenscroft J (2004). Thyroid function in relation to burden of PCBs, *p,p*′-DDE, HCB, mirex and lead among Akwesasne Mohawk youth: a preliminary study. Environ Toxicol Pharmacol.

[b52-ehp0115-001780] Schisterman EF, Whitcomb BW, Buck Louis GM, Louis TA (2005). Lipid adjustment in the analysis of environmental contaminants and human health risks. Environ Health Perspect.

[b53-ehp0115-001780] Soechitram SD, Athanasiadou M, Hovander L, Bergman Å, Sauer PJJ (2004). Fetal exposure to PCBs and their hydroxylated metabolites in a Dutch cohort. Environ Health Perspect.

[b54-ehp0115-001780] Steuerwald U, Weihe P, Jorgensen PJ, Bjerve K, Brock J, Heinzow B (2000). Maternal seafood diet, methyl-mercury exposure, and neonatal neurologic function. J Pediatr.

[b55-ehp0115-001780] Sunyer J, Torrent M, Muñoz-Ortiz L, Ribas-Fitó N, Carrizo D, Grimalt J (2005). Prenatal dichlorodiphenyldichloroethylene (DDE) and asthma in children. Environ Health Perspect.

[b56-ehp0115-001780] Szpir M (2006). New thinking on neurodevelopment. Environ Health Perspect.

[b57-ehp0115-001780] Takser L, Mergler D, Baldwin M, de Grosbois S, Smargiassi A, Lafond J (2005). Thyroid hormones in pregnancy in relation to environmental exposure to organochlorine compounds and mercury. Environ Health Perspect.

[b58-ehp0115-001780] Walker JB, Seddon L, McMullen E, Houseman J, Tofflemire K, Corriveau A (2003). Organochlorine levels in maternal and umbilical cord blood plasma in Arctic Canada. Sci Total Environ.

[b59-ehp0115-001780] Walkowiak J, Wiener JA, Fastabend A, Heinzow B, Krämer U, Schmidt E (2001). Environmental exposure to polychlorinated biphenyls and quality of the home environment: effects on psychodevelopment in early childhood. Lancet.

[b60-ehp0115-001780] Wang SL, Su PH, Jong SB, Guo YL, Chou WL, Päpke O (2005). *In utero* exposure to dioxins and polychlorinated biphenyls and its relations to thyroid function and growth hormone in newborns. Environ Health Perspect.

[b61-ehp0115-001780] Williams FLR, Simpson J, Delahunty C, Ogston SA, Bongers-Schokking JJ, Murphy N (2004). Developmental trends in cord and postpartum serum thyroid hormones in preterm infants. J Clin Endocrinol Metab.

[b62-ehp0115-001780] Zoeller RT, Robertson LW, Hansen LG (2001). Polychlorinated biphenyls as disruptors of thyroid hormone action. PCBs: Recent Advances in Environmental Toxicology and Health Effects.

[b63-ehp0115-001780] Zoeller RT (2005). Environmental chemicals as thyroid hormone analogues: new studies indicate that thyroid hormone receptors are targets of industrial chemicals?. Mol Cell Endocrinol.

